# Inflammatory Response and Exosome Biogenesis of Choroid Plexus Organoids Derived from Human Pluripotent Stem Cells

**DOI:** 10.3390/ijms24087660

**Published:** 2023-04-21

**Authors:** Laureana Muok, Chang Liu, Xingchi Chen, Colin Esmonde, Peggy Arthur, Xueju Wang, Mandip Singh, Tristan Driscoll, Yan Li

**Affiliations:** 1 Department of Chemical and Biomedical Engineering, FAMU-FSU College of Engineering, Florida State University, Tallahassee, FL 32310, USA; 2 College of Pharmacy and Pharmaceutical Sciences, Florida A&M University, Tallahassee, FL 32307, USA; 3 Department of Materials Science and Engineering, University of Connecticut, Storrs, CT 06268, USA

**Keywords:** human pluripotent stem cells, choroid plexus organoids, Wnt signaling, inflammatory response, extracellular vesicles

## Abstract

The choroid plexus (ChP) is a complex structure in the human brain that is responsible for the secretion of cerebrospinal fluid (CSF) and forming the blood–CSF barrier (B-CSF-B). Human-induced pluripotent stem cells (hiPSCs) have shown promising results in the formation of brain organoids in vitro; however, very few studies to date have generated ChP organoids. In particular, no study has assessed the inflammatory response and the extracellular vesicle (EV) biogenesis of hiPSC-derived ChP organoids. In this study, the impacts of Wnt signaling on the inflammatory response and EV biogenesis of ChP organoids derived from hiPSCs was investigated. During days 10–15, bone morphogenetic protein 4 was added along with (+/−) CHIR99021 (CHIR, a small molecule GSK-3β inhibitor that acts as a Wnt agonist). At day 30, the ChP organoids were characterized by immunocytochemistry and flow cytometry for TTR (~72%) and CLIC6 (~20%) expression. Compared to the −CHIR group, the +CHIR group showed an upregulation of 6 out of 10 tested ChP genes, including *CLIC6* (2-fold), *PLEC* (4-fold), *PLTP* (2–4-fold), *DCN* (~7-fold), *DLK1* (2–4-fold), and *AQP1* (1.4-fold), and a downregulation of *TTR* (0.1-fold), *IGFBP7* (0.8-fold), *MSX1* (0.4-fold), and *LUM* (0.2–0.4-fold). When exposed to amyloid beta 42 oligomers, the +CHIR group had a more sensitive response as evidenced by the upregulation of inflammation-related genes such as *TNFα*, *IL-6*, and *MMP2*/*9* when compared to the −CHIR group. Developmentally, the EV biogenesis markers of ChP organoids showed an increase over time from day 19 to day 38. This study is significant in that it provides a model of the human B-CSF-B and ChP tissue for the purpose of drug screening and designing drug delivery systems to treat neurological disorders such as Alzheimer’s disease and ischemic stroke.

## 1. Introduction

Located in each ventricle of the brain, the choroid plexus (ChP) is a highly vascularized tissue. It is responsible for the secretion of cerebrospinal fluid (CSF) and forming the blood–CSF barrier (B-CSF-B). Various studies have emerged highlighting the importance of CSF in the central nervous system (CNS) [1]. Despite these discoveries, the ChP is a relatively understudied tissue in neuroscience. Some of the reasons for this include historical perceptions regarding the roles of ChP, experimental issues, and a lack of clinical interest [1].

Recently there has been an increased interest in understanding the role that the ChP and CSF play in regulating the structure and function of the CNS, with the view that the ChP could be a therapeutic target [2]. This interest has led to new hypotheses on the impact that the ChP and CSF have on health and disease, as well as new therapeutic approaches using pluripotent stem cell technologies. For example, several studies have found a link between the ChP–CSF system and Alzheimer’s disease (AD) as well as SARS-CoV-2 neurotropism [3]. The observations from these studies have suggested that by harnessing CSF, it may be possible to tailor personalized therapeutic approaches for detecting, diagnosing, and treating neurodegeneration such as AD. In addition to AD, other studies have shown the potential for the ChP to aid in treating hydrocephalus, cancer, and other neurodegenerative diseases [2]. To model these types of neurological disorders, a variety of recent stem cell models have been developed and characterized [4,5,6,7]. Furthermore, human ChP organoids have been recently established as an in vitro model for testing drug permeability through the ChP [8]. Using these types of organoid models, the development of methods for overcoming current obstacles, such as the blood–brain barrier (BBB) or B-CSF-B, may be possible [2].

Due to the complexity of the human brain, many brain disorders cannot be modelled in traditional model organisms. To tackle this issue, Lancaster et al. developed a human pluripotent stem cell (hPSC)-derived 3D brain organoid system that included both human embryonic stem cells (hESCs) and human-induced pluripotent stem cells (hiPSCs) [5,9,10]. That work focused on improving growth conditions and providing the environment necessary for intrinsic cues to influence development [11]. This was accomplished by maintaining neuroectodermal tissues in 3D culture embedded in Matrigel droplets. In addition, this study highlighted the importance of CHIR99021 (CHIR) and bone morphogenetic protein (BMP) 4 in ChP differentiation [8]. CHIR is a small molecule that is known to activate the canonical Wnt/β-catenin pathway by acting as a glycogen synthase kinase 3 beta (GSK-3β) inhibitor [12,13]. When hPSCs grown in 3D culture were exposed to CHIR alone, it resulted in cortical-hem-like tissues. When they were exposed to BMP4 alone, partially induced choroid-plexus-like tissue was observed. The final condition of using CHIR and BMP4 showed a strong dorsalizing activity and dominantly induced choroid-plexus-like tissues [8]. However, the inflammatory response of the derived ChP organoids to the amyloid beta 42 oligomers in order to mimic AD pathology has not been well investigated.

ChP-secreted extracellular vesicles (EVs) play an important role in AD pathology [14]. The ChP has been identified as a major source of the cerebrospinal fluid’s (CSF) EVs, which contain brain-specific proteins as well as ChP-associated proteins such as TTR [15]. The B-CSF-B interface, formed by the ChP epithelial cells, has been reported to release an increased amount of EVs into the CSF in response to peripheral inflammation and thus spread the inflammation to the CNS [16,17]. For example, Aβ oligomers-induced ChP-derived EVs (containing IGFBP3, ICAM-1, SMPD1 proteins etc.) have been reported to induce proinflammatory effects in cortical cell cultures. A proteome analysis of these EVs revealed the increased expression of several proinflammatory proteins, including the complement protein C3, as well as monocyte chemoattractant protein-1 (MCP1), IL-6, and CCL5 [16,17]. For a potential gain or loss of function analysis, GW4869 is a specific, noncompetitive inhibitor of nSMase 2, which can block exosome biogenesis and release [18]. Using GW4869 to block the amyloid beta (Aβ)42 oligomer-induced ChP-EV secretion is a potential approach to prevent cognitive decline with AD pathology. However, the EV biogenesis of ChP organoids has not been well investigated.

Our previous studies evaluated the effects of Wnt signaling in modulating YAP and Notch activities, as well as the microRNAs that modulate Wnt signaling in cortical and cerebellar organoids patterning from hiPSCs [19,20,21,22,23]. In this study, the inflammatory response to Aβ42 oligomer stimulation was evaluated for the ChP organoids derived from hiPSCs in the presence or absence of CHIR. Since both lineage specification of hiPSCs and the 3D culture environment can affect cell–cell communication through changes in EV secretion [24,25,26], the influence of the Wnt activation on the ChP-derived EV biogenesis and production was investigated. To date, no study has assessed the inflammatory response and the EV biogenesis of hiPSC-derived ChP organoids. This work is significant in that it characterizes the inflammatory response and EV biogenesis of ChP organoids, which are critical components for modeling the human B-CSF-B. This provides a testbed for future drug screening and delivery system design, to improve treatment of neurological disorders.

## 2. Results

### 2.1. ChP Organoid Differentiation and Characterization

**Figure 1:**
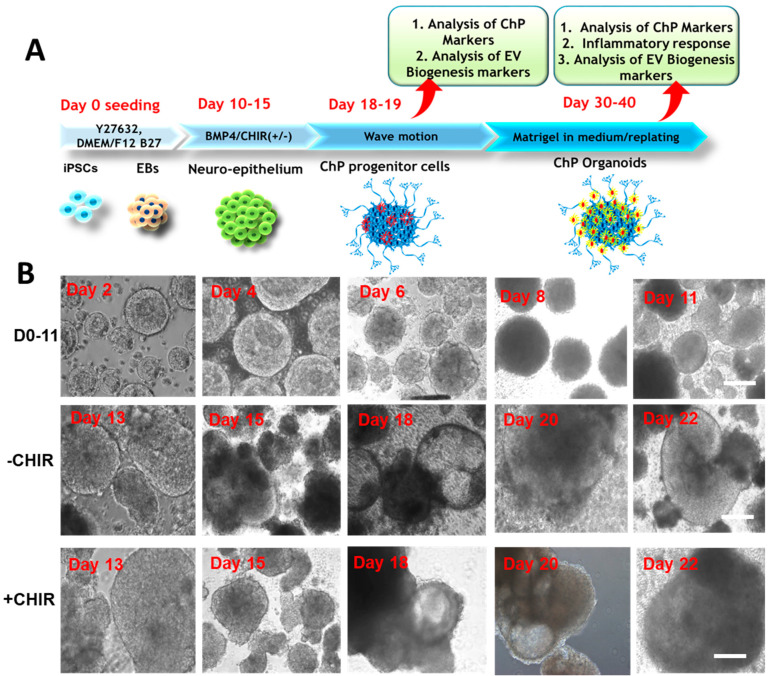
**ChP organoid differentiation from hiPSCs.** (**A**) Illustration of ChP differentiation; (**B**) the ChP organoid morphology for CHIR+/− conditions during the differentiation. Scale bar: 200 µm. BMP: bone morphogenetic protein; EBs: embryoid bodies. EV: extracellular vesicles.

**Figure 2:**
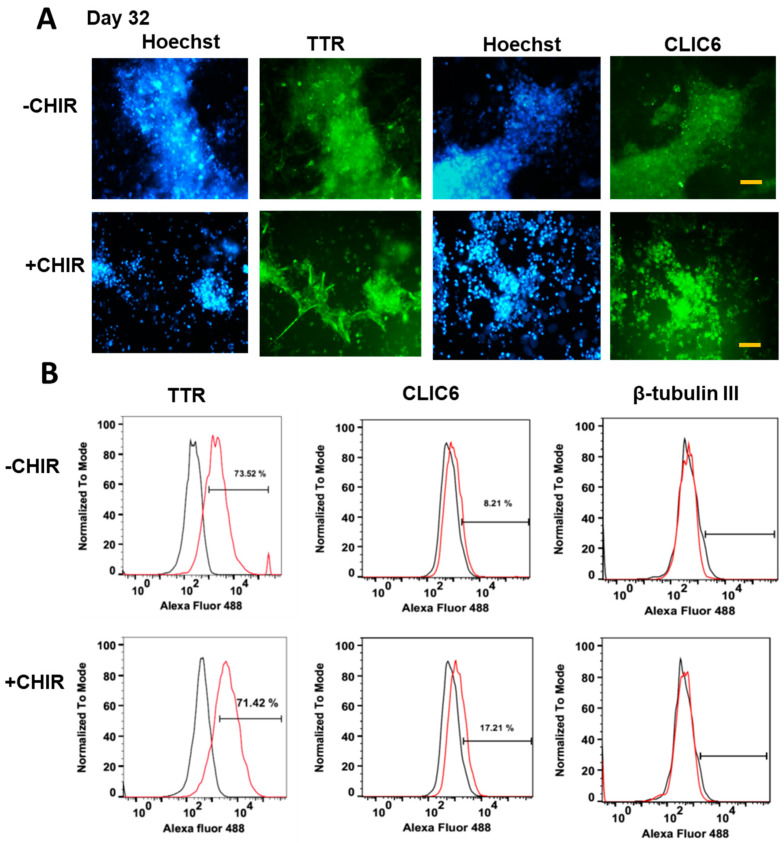
**Expression of ChP markers for CHIR+/− conditions.** (**A**) Immunostaining of ChP markers at day 32. Scale bar: 50 µm. (**B**) Flow cytometry histograms of ChP markers for CHIR+/− conditions. The assays were performed at day 30. Black line: negative control. Red line: marker of interest. TTR (transthyretin) and CLIC6 (chloride intracellular channel 6) are ChP markers. CHIR(+/−) indicates treatment with CHIR99021.

During the ChP organoid development, the spheroids were imaged every 2–3 days prior to media change to assess the growth and morphology of the ChP organoids (Figure 1A). The spheroids increased in size from ~300 µm at day 3 to ~1200 µm after day 22, and the size was comparable for the two groups (CHIR+/−) (Supplementary Figure S1). However, the CHIR+ groups in general had 2–3-fold more spheroids than the CHIR− group by day 30, possibly due to the stimulated proliferation by Wnt activation, based on the amount of total mRNA isolated (1.82 ± 0.47-fold for CHIR+/CHIR− ratios) (Supplementary Figure S2). The ChP organoids were enriched with cuboidal epithelium. Cysts containing a colorless fluid (fluid filled compartments), a notable feature of ChP organoids, were visible after day 18 of development for both conditions (Figure 1B). On day 32 of development, immunostaining was performed for the intracellular markers of replated ChP organoids, CLIC6 and transthyretin (TTR) (Figure 2A). The positive expression levels of CLIC6 and TTR were observed for both conditions. Since the nuclear staining showed some granules, a LIVE/DEAD assay was performed, and the dead cells were present in the replated organoids (Supplementary Figure S3). On day 30, flow cytometry was performed for the dissociated ChP organoid cells in order to quantify the ChP markers TTR and CLIC6 (Figure 2B and Supplementary Figure S4). For TTR, about 71–74% of expression was observed, while CLIC6 displayed 8–17% of expression (17.2% vs. 8.2% for CHIR+ vs. CHIR− group, respectively). Compared to the CHIR− condition, the CHIR+ condition had a ~10% higher CLIC6 expression, while the TTR expression was comparable. The general neuronal marker β-Tubulin III showed little expression for both CHIR+/− conditions, indicating that the derived ChP organoids contained few neuronal cells.

**Figure 3:**
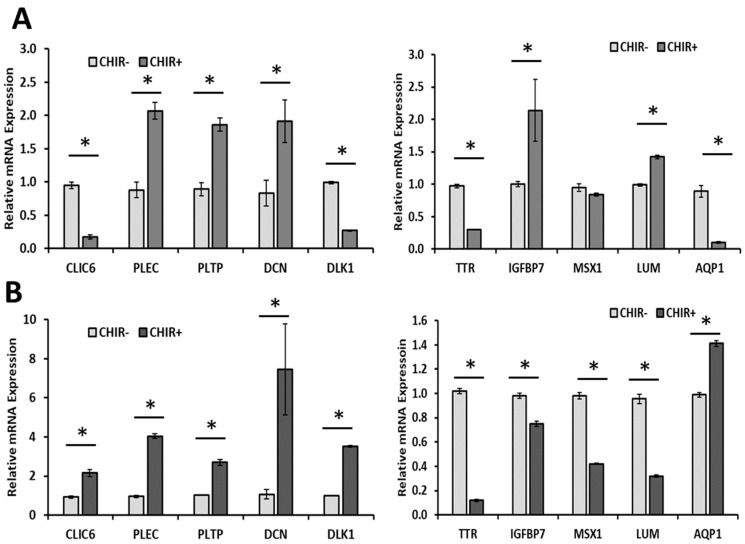
**mRNA expression of ChP markers at days 18 and 30 for CHIR+/− conditions.** mRNA expression was determined by RT-qPCR. (**A**) Day 18; (**B**) day 30 of differentiation. N = 3. * indicates *p* < 0.05. CHIR(+/−) indicates treatment with CHIR99021.

On day 18 and day 30, the relative mRNA expression levels of various ChP markers in addition to CLIC6 and TTR were determined (Figure 3) [8]. *PLEC* encodes plectin, an intermediate-filament-binding protein. *PLTP* encodes a phospholipid transfer protein. *DCN* encodes decorin, a small cellular or pericellular matrix proteoglycan closely related to biglycan. *DLK1* encodes a transmembrane protein delta-like noncanonical Notch ligand 1, regulating cell–cell interactions. *AQP1* encodes aquaporin 1, a widely expressed water channel protein. *IGFBP7* encodes insulin-like growth-factor-binding protein 7, whose major function is to regulate the availability of insulin-like growth factors (IGFs) and modulate IGF binding to its receptors. *MSX1* encodes Msh homeobox 1, an early regulatory factor involved in ChP development. *LUM* encodes lumican, a proteoglycan member in the small leucine-rich proteoglycan (SLRP) family. These markers should be present in the ChP organoids [8]. It was observed that, when compared to the CHIR− group, the CHIR+ group showed an upregulation of 5 out of 10 genes (Figure 3A). At day 30, the CHIR+ group showed an upregulation of 6 out of 10 genes compared to the CHIR− group: *CLIC6* (2-fold), *PLEC* (4-fold), *PLTP* (2–4 fold), *DCN* (~7-fold), *DLK1* (2–4-fold), and *AQP1* (1.4-fold), and down-regulation of *TTR* (0.1-fold), *IGFBP7* (0.8-fold), *MSX1* (0.4-fold), and *LUM* (0.2–0.4-fold), all with *p* < 0.05 (Figure 3B). Compared to day 18, the trend of *PLEC*, *PLTP*, *DCN*, *TTR*, and *MSX1* in the comparison of CHIR+ to CHIR− condition remained the same at day 30. *CLIC6*, *DLK1*, and *AQP1* all displayed an upregulation while *IGFBP7* an *LUM* were downregulated at day 30 compared to day 18.

### 2.2. The Immune Response to Aβ42 Oligomers

**Figure 4:**
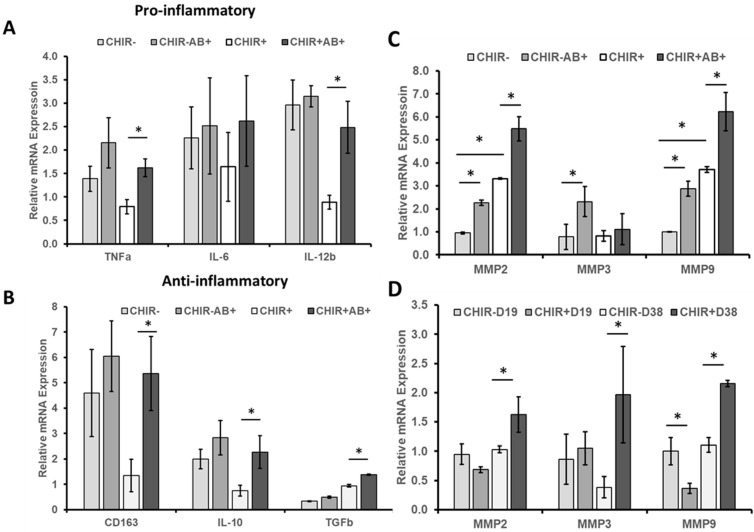
**Inflammatory response to Aβ42 oligomer stimulation for ChP organoids of CHIR+/− conditions**. mRNA expression determined by RT-qPCR for (**A**) proinflammatory markers (day 33); (**B**) anti-inflammatory markers (day 33); these markers were relative to the CHIR+ condition. (**C**) MMP2, MMP3, and MMP9 expression affected by Aβ42 oligomers (day 33); (**D**) MMP2, MMP3, and MMP9 expression affected by CHIR at days 19 and 38. N = 3. * indicates *p* < 0.05. The MMP2, MMP3, and MMP9 expression was relative to the CHIR−D19 condition. CHIR(+/−) indicates treatment with CHIR99021, D19 and D38 indicates days 19 and 38, AB(+/−) indicates treatment with amyloid β 42 oligomers. The CHIR+ group is more responsive than the CHIR− group.

Aβ42 oligomers can induce a proinflammatory response, and the accumulation of Aβ42 oligomers in the brain has been proposed to be an early toxic event in the pathogenesis of Alzheimer’s disease [27,28]. To assess these effects in our ChP organoid model, Aβ42 oligomers were added to day 30 CHIR(+/−)-derived ChP organoids for three days. The proinflammatory response was evaluated by the expression of *TNFα*, *IL-6*, and *IL-12β*. The anti-inflammatory response was evaluated by the expression of *TGFβ*, *IL-10*, and *CD163* (Figure 4A,B). Higher levels of both pro- and anti-inflammatory markers to the same stimulus have previously been reported in the literature [29], indicating a more activated (but not necessarily more inflamed) inflammatory system. There were no significant changes in these six markers when adding Aβ42 oligomers to the CHIR− group. For the CHIR+ group, *TNFα* (1.5-fold) and *IL-12β* (2.5-fold) were significantly upregulated (*p* < 0.05) after adding Aβ42 oligomers, while *IL-6* had no significant change. *CD163* (5-fold), *IL-10* (2-fold), and *TGFβ* (1.5-fold) were all significantly upregulated (*p* < 0.05) after adding Aβ42 oligomers. Overall, the response of the CHIR+ group to the Aβ42 oligomer stimulation was more sensitive than the CHIR− group in terms of expression of pro- and anti- inflammatory markers.

Aβ42 oligomers can activate matrix metalloproteinases (MMPs), therefore the expression of *MMP2*, *MMP3*, and *MMP9* was determined (Figure 4C,D). For CHIR− conditions, Aβ42 oligomers upregulated the *MMP2*, *MMP3*, and *MMP9* expression by 2–3-fold (*p* < 0.05). The CHIR+ condition upregulated *MMP2* and *MMP9* by 3–4-fold (*p* < 0.05), but not MMP3. Aβ42 oligomers further upregulated the *MMP2* and *MMP9* expression by 5.5–6.2-fold (*p* < 0.05), while *MMP3* expression remained at a similar level to the CHIR− condition without Aβ42 oligomers. To further separate the effects of Aβ42 oligomers from the CHIR treatment, day-19 and day-38 cells of CHIR+/− were assessed. At day 19, the CHIR treatment did not upregulate *MMPs*, while at day 38, the CHIR treatment upregulated *MMP2*, *3*, and *9* by 1.6–3.2-fold (*p* < 0.05). Together, the response of the CHIR+ group to the Aβ42 oligomers stimulation was more sensitive than that of the CHIR− group in terms of MMP activation.

### 2.3. Extracellular Vesicle Biogenesis of ChP Organoids

**Figure 5:**
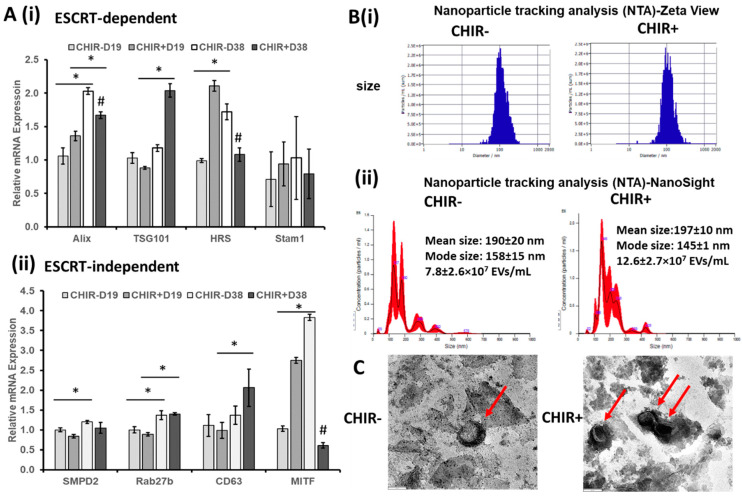
**Extracellular vesicle biogenesis of ChP organoids with CHIR+/− conditions.** (**A**) mRNA expression determined by RT-qPCR for (i) ESCRT-dependent EV biogenesis markers; (ii) ESCRT-independent EV biogenesis markers; N = 3. * indicates *p* < 0.05 for the compared conditions, and # indicates *p* < 0.05 compared to the CHIR− condition at the same time point. CHIR(+/−) indicates treatment with CHIR99021 (3 µM), D19 and D38 indicates days 19 and 38 during differentiation. (**B**) Nanoparticle tracking analysis (NTA) for the isolated ChP organoid-EVs; (i) particle size distribution and zeta potential by ZetaView; (ii) particle size distribution by NanoSight. (**C**) Transmission electron microscopy images showing exosome morphology; scale bar: 100 nm.

Organoid models can provide a method for the in vitro generation of tissue-specific EVs, and many have been shown to have a therapeutic potential [24]. Additionally, ChP-EVs play a critical role in neurodegenerative disease pathophysiology [16]. To assess the EVs produced in this ChP organoid model, RT-qPCR was performed on days 19 and 38 for the expression of EV biogenesis markers, including both the endosomal sorting complex required for transport (ESCRT)-dependent and -independent biogenesis, in the derived ChP organoids (Figure 5A) [30,31]. For the majority of the EV biogenesis markers (except *Stam1*), there was an increase in general with ChP tissue development from day 19 to day 38. CHIR+ upregulated the ESCRT-dependent marker *TSG101* (2-fold) and the ESCRT-independent marker *CD63* (2-fold) at day 38 compared to day 19 (*p* < 0.05). The ESCRT-dependent markers *Alix* (1.4-fold) and *HRS* (2-fold) and the ESCRT-independent marker *MITF* (2.8-fold) were also upregulated (*p* < 0.05) at day 38 compared to day 19 for the CHIR− group. For the CHIR+ cells, there was an upregulation for *TSG101*, but a decrease for *MITF*, *HRS*, and *Alix* at day 38 compared to the CHIR− group but not at day 19, showing the differential expression of these markers regulated by Wnt activation.

EVs secreted by CHIR(+/−)-derived ChP organoids were isolated using the ExtraPEG method along with a differential ultracentrifugation (Supplementary Figure S5). An NTA analysis using ZetaView showed that the derived EVs had an average size of 105.2 nm and 105.0 nm (43.5 nm as span) for the CHIR+ and CHIR− groups (Figure 5Bi), respectively. The zeta potential was −32.88 ± 1.14 mV and −36.10 ± 1.06 mV, respectively, for CHIR+ and CHIR− groups (Supplementary Figure S6). Using NanoSight, the derived EVs had an average mean size of 190 ± 20 nm and 197 ± 10 nm and an average mode size of 158 ± 15 nm and 145 ± 1 nm for the CHIR+ and CHIR− groups, respectively. The mode size can eliminate the interference from protein aggregation and should be more accurate than the mean size. The particle concentration was higher with 12.6 ± 2.7 × 10^7^ particles/mL (1.62-fold) for the CHIR+ group than the CHIR− group (7.8 ± 2.6 × 10^7^ particles/mL) when the same quantity of media was collected for EV isolation (Figure 5Bii). The EV number was also normalized to the mRNA amount for each condition (Supplementary Table S1), which indicated that the higher EV number in the CHIR+ condition was probably due to the higher cell number. TEM images indicated a typical cup-shaped exosome morphology, which confirmed the presence of exosomes (Figure 5C).

## 3. Discussion

Prior research has shown that the ChP epithelium is derived from neuroepithelial tissue found in the telencephalon, diencephalon, and hindbrain regions of the brain. It has been reported that the portions of the telencephalon that are responsible for the ChP epithelium production are enriched in genes from the BMP and Wnt protein families [32]. A recent study investigated the impact of Wnt signaling on the ChP epithelium’s development [33], using embryonic mouse and human models as well as hESCs to derive organoids. It was observed that the canonical Wnt signaling pathway was activated in the developing choroid plexus of both embryonic mouse and human brains. It was also hypothesized that the regulation of the Wnt/β-catenin pathway was finely tuned in vivo. This was due to the finding that a loss or gain of Wnt/β-catenin function led to a disruption in the ChP epithelium’s development [33]. During embryonic brain development, it has been observed that multiple BMP family members are present [34]. BMP4, specifically, is most prominently found in the areas that eventually develop into the ChP. Additionally, BMP4 has been shown to induce ChP epithelial cell fate [35]. Consistently, a study on embryonic mouse brains showed that BMP signaling and the BMPR1A receptor were required for ChP formation [35].

Canonical Wnt activation has been established as a critical component for ChP organoid development, but the constitutive activation of canonical Wnt signaling disrupts the choroid plexus epithelial fate [33]. The addition of CHIR at an earlier time point (days 5–10) may more easily induce hPSCs to differentiate into mesoderm cells such as cardiovascular cells. The addition of CHIR at a later time point (days 15–20) is usually used to pattern neural tissue [36,37,38]. Therefore, the CHIR treatment was only used during days 10–15 of the ChP organoid development. Wnt activation upregulated 6 out of the 10 ChP markers (e.g., *CLIC6*, *PLEC*, *PLTP*, *DCN*, *DLK1*, and *AQP1*) assessed in this study. The cell population may not be the same under CHIR− and CHIR+ conditions (also shown as differences in the efficiency of differentiation). Therefore, the activation of the Wnt signaling pathway may lead to different cell populations with differential cellular responses. The ChP marker expression was also examined in microglia-containing forebrain organoids, based on mRNA-seq data from our previous work (Supplementary Table S2) [39]. The majority of the ChP markers were also upregulated in 3D forebrain organoids. The ChP tissue development as 3D organoids provides in vivo-like cell–cell interactions that are likely important for gaining ChP tissue identity during differentiation from hiPSCs. The ChP organoids derived in this study should have a telencephalic origin with a lateral ventricle (not third and fourth ventricle) identity [8]. The absence of β-tubulin III expression confirmed the absence of neuronal cells in the derived ChP organoids, while abundant neuronal cells are usually observed in forebrain (which requires Wnt inhibition) and cerebellar organoids (which requires Wnt activation) [22,40,41,42]. While the previous study used the derived ChP organoids to test the drug permeability of the B-CSF-B barrier [8], no study has yet assessed the inflammatory response and the EV biogenesis of the hiPSC-derived ChP organoids.

The B-CSF-B barrier in the ChP plays a crucial role in the spreading of inflammatory reactions in the CNS, and the inflammatory response of ChP cells contributes to the pathogenesis and progression of various neurological disorders such as Alzheimer’s disease [43]. There is evidence that the ChP can selectively clean Aβ from the CSF, while the underlying mechanism is unclear [44]. Treatment with Aβ oligomers has been reported to promote the proliferation and differentiation of neuronal progenitor cells cocultured with ChP cells, while the survival of newly formed neurons was decreased [45]. Pretreating ChP epithelial cells with a specific inhibitor of mitogen-activated protein kinase signaling can diminish Aβ-induced neuronal proliferation. Little is known about transport and metabolic responses by the ChP to the disrupted homeostasis due to CNS Aβ. When there was ChP dysfunction, the increased expression of the choroidal Aβ transporters was observed, including the low-density lipoprotein receptor-related protein 1 (LRP1) and the receptor for advanced glycation end product (RAGE) [46]. As one of the pathological responses, a thickening of the epithelial basal membrane and a collagen IV deposition were observed around capillaries in the ChP. hiPSC-derived ChP models could be used for the relevant pathological studies.

In this study, CHIR+-derived ChP organoids were more responsive to Aβ42 oligomer stimulation than the CHIR− condition, as shown by the upregulation of proinflammatory and anti-inflammatory markers as well as *MMP2*/*9*, which is likely of physiologic relevance for disease modeling and drug screening investigations. This suggests that Wnt signaling plays a part in regulating the inflammatory response within the ChP tissue, consistent with previous observations of the role of Wnt signaling in neuroinflammation [47,48]. Aβ42 oligomers induce neuroinflammation in the ChP resulting in a leakage of the B-CSF-B barrier by activating MMPs and TNF receptor-1 signaling [49]. Therefore, the *MMP2*, *MMP3*, and *MMP9* expression of ChP organoids in response to Aβ42 oligomers was measured in this study. Consistently, both CHIR− and CHIR+ groups showed the upregulation of *MMP2* and *MMP9* with the Aβ42 oligomer stimulation, but the CHIR+ group was more responsive. In the future, more pathologically relevant markers can be examined.

This study also investigated the EV secretion from ChP organoids under normal conditions (not by Aβ42 oligomer stimulation), as Aβ42 oligomer stimulation can significantly alter the composition of the EV proteome secreted by ChP cells [16]. The EV biogenesis ability was increased with the ChP organoid development time. A similar observation of EV secretion from retinal organoids derived from hiPSCs was observed in our previous work [50]. In vitro systems for generating healthy ChP-secreted EVs are also of potential therapeutic relevance and may be able to reverse the pathology of neurodegeneration. For example, young CSF (containing ChP-secreted EVs) can restore the oligodendrogenesis and memory in aged mice [51]. Through NTA, there was no significant difference in EV size and zeta potential. The particle concentration for the CHIR+ condition was higher than that of the CHIR− condition, which may be related to the higher cell numbers (i.e., higher proliferation) promoted by the Wnt activation. An EV cargo analysis (proteomics for protein cargo and miRNA profiling for miRNA cargo) may need to be performed in the future to assess the changes in EV composition. The EV cargo medications such as loading with curcumin may be examined in the future in order to modulate the neuro-inflammation in AD.

In addition to AD, the ChP can be a site of damage in ischemic stroke and display alterations (e.g., neurogenesis, neural progenitor cell migration, leukocyte infiltration) in response to the associated brain injury [52]. The ChP has protective effects in brain injury, producing neurotrophic/neuroprotective factors such as fibroblast growth factor 2, insulin-like growth factor 2, vascular endothelial growth factor, transforming growth factor β, etc. The resulting hypoxia/ischemia can cause cell death in the ChP as well as a disruption of the B-CSF-B barrier, and the ChP is selectively vulnerable. The organoids described here can provide a valuable in vitro model to study the ChP response to hypoxia/ischemia in stroke.

## 4. Materials and Methods

### 4.1. Undifferentiated hiPSC Culture

The iPSK3 cell line was obtained by transfecting human foreskin fibroblasts with plasmid DNA encoding reprogramming factors octamer-binding transcription factor 4 (OCT4), NANOG, SRY-box transcription factor 2 (SOX2), and LIN28 (kindly provided by Dr. Stephen Duncan, Medical College of Wisconsin) [53,54]. The cells were cultured in mTeSR Plus serum-free medium (StemCell Technologies, Inc., Vancouver, BC, Canada) on a growth-factor-reduced Geltrex-coated surface (Life Technologies, Carlsbad, CA, USA) [41,55]. Before seeding, 1 mL of 1% Geltrex was added to a 6-well attachment plate. Coating was done at 37 °C for at least one hour. The coating solution was then removed, and 1.5 × 10^6^ cells were seeded in 3 mL of fresh mTeSR^TM^ Plus medium with 10 µM ROCK inhibitor Y-27632 (dilution, 1:1000). After 24 h, Y-27632 was removed and the media were replaced with fresh mTeSR^TM^ Plus media. The media were changed daily for 4–5 days until the cells were ready to be passaged again.

### 4.2. ChP Organoid Differentiation

ChP differentiation was performed using a modified protocol based on the literature [8]. HiPSCs were seeded onto a 24-well ultralow attachment plate at 3 × 10^5^ cells/well, in 1 mL of fresh Dulbecco’s Modified Eagle Medium/Nutrient Mixture F-12 (DMEM/F-12) plus 2% B27 serum-free supplement (Cat# 17504044, Life Technologies under ThermoFisher, Carlsbad, CA, USA) in the presence of 10 µM ROCK inhibitor Y-27632 (Sigma-Aldrich, Burlington, MA, USA). The cells were then incubated at 37 °C for 24 h. On day 1, the medium was exchanged with DMEM/F12-B27 (Y27632 was removed); going forward, the medium was changed every other day. Starting on day 10, 3 μM CHIR99021 (CHIR) (Sigma-Aldrich) and 20 ng/mL BMP4 (Peprotech, Cranbury, NJ, USA) were added to the DMEM/F12-B27 media. This was repeated every other day until day 15, when the medium was reverted back to DMEM/F12-B27 only. Additionally, the organoids were moved to an incubator with a shaker and fed every 3–4 days. Starting on day 30, dissolved Matrigel (1:50) (Corning) was added to the maturation media. The derived ChP organoids were characterized between days 18–20 and days 30–40. The cultures less than 30 days were referred as spheroids, and the cultures longer than 30 days were referred as organoids. 

### 4.3. Image Analysis of Aggregate Size

For aggregate size distribution, images were analyzed using ImageJ software (National Institutes of Health, Bethesda, MD, USA) to calculate aggregate diameters. Specifically, the captured images were converted to binary images using ImageJ (http://rsb.info.nih.gov/ij, accessed on 13 April 2023) and analyzed with the “analyze particles” tool, which output the Feret’s diameter of each aggregate, providing the average size of the aggregates (n = 5–10).

### 4.4. Immunocytochemistry (ICC)

After 25–35 days, the organoids were replated. Briefly, 150 µL of 1% Geltrex (or Matrigel) was added to a 24-well attachment plate at 37 °C for at least one hour. The coating solution was then removed, and the organoids were seeded with 1 mL of DMEM/F12 medium per well (1:2 ratio). For ICC, the medium was removed, cells were washed with phosphate buffered saline (PBS), and then 4% paraformaldehyde (PFA) was added for 20–30 min at room temperature (RT). The cells were then washed again with PBS. Permeabilization was carried out by adding 100% cold ethanol. After incubating for 5 min at RT, the cells were washed with PBS. Blocking was performed with 5% fetal bovine serum (FBS) in PBS for 30 min at RT. For immunostaining, cells were incubated with TTR, CLIC6, and beta-tubulin III primary antibody (Supplementary Table S3) in a staining buffer (1% FBS in PBS) for one hour at RT. The cells were then washed with PBS and stained with secondary antibody diluted in PBS in the dark. After incubating for one hour at RT, the cells were washed one last time with PBS. The cell nuclei were stained with Hoechst 33342 (blue) and imaged via an epifluorescence microscope (Olympus IX70).

### 4.5. LIVE/DEAD Assay 

The cells were evaluated for viability using the Live/Dead™ staining kit (Molecular Probes) according to the manufacturer’s protocol. The replated spheroids were washed with PBS and then incubated in DMEM-F12 containing 2 μM calcein-AM (green) and 0.8 μM ethidium homodimer I (red) for 20–25 min at room temperature and protected from light. The images were taken under a fluorescent microscope (Olympus IX70, Melville, NY, USA).

### 4.6. Flow Cytometry

ChP organoids were dissociated by 0.25% Trypsin/EDTA, and the single cells in suspension were fixed with 4% PFA for 15 min, spun at 300× *g* for 5 min to remove supernatant, and then resuspended in PBS at 10^7^ cells per mL. Nine volumes of cold 100% methanol were then added, and the cells were incubated on ice for 15–30 min to permeabilize. Cells were spun at 300× *g* for 5 min to aspirate the supernatant, resuspended in blocking buffer, and incubated at 4 °C for 15 min. Primary antibodies (Supplementary Table S3) prepared in a staining buffer (SB) were then added to the cells and incubated at 4 °C for one hour. After incubation, the cells were washed with an SB and then spun at 300× *g* for 5 min. While the cells were spinning, the secondary antibody was prepared in an SB in the dark. After washing, the secondary antibody was added and incubated at 4 °C for 30–60 min in the dark. After incubation, the cells were washed with 2 mL of SB and spun at 300× *g* for 5 min. The cells were resuspended in 150 μL of staining buffer. The samples were analyzed with a BD FACSCanto™ II flow cytometer (Becton Dickinson, Franklin Lakes, NJ, USA) and compared against isotype controls using FlowJo software.

### 4.7. Quantitative Reverse Transcription-Polymerase Chain Reaction (RT-qPCR) Analysis

Total RNA was isolated from organoid samples using the RNeasy Mini Kit (Qiagen, Valencia, CA, USA) according to the manufacture’s protocol, followed by a treatment with the DNA-Free RNA Kit (Zymo, Irvine, CA, USA). Reverse Transcription was carried out using 2 μg of total RNA, anchored oligo-dT primers, and Superscript III (Invitrogen, Carlsbad, CA, USA, according to the manufacturer’s protocol). The primers specific to the targeted gene were designed using Primer-BLAST software (NIH Database) (Supplementary Table S4). The gene β-actin was used as an endogenous control for the normalization of expression levels. RT-qCR reactions were performed on an ABI7500 instrument (Applied Biosystems, Foster City, CA, USA), using the SYBR Green PCR Master Mix (Applied Biosystems). The amplification reactions were performed as follows: 2 min at 50 °C; 10 min at 95 °C; and 40 cycles of 95 °C for 15 s; 55 °C for 30 s; and 68 °C for 30 s. The Ct values of the target genes were first normalized to the Ct values of the endogenous control β-actin. The corrected Ct values were then compared for the treatment conditions to the untreated control. Fold changes in gene expression were calculated using the comparative Ct method, $2^{{-(∆C}_{t\;treatment}-{∆C}_{t\;control})}$, to obtain the relative expression levels.

### 4.8. Immune Response to Amyloid Beta 42 Oligomer Stimulation

To prepare oligomers of Aβ(1-42) peptide, biotinylated Aβ(1-42) (Bachem) was fully dissolved at 0.5 mg/mL in hexafluor-2-propanole (HFIP, Sigma) [56,57]. Then, 10 μL of HFIP Aβ(1-42) solution was dispensed into a siliconized Snap-Cap microtube, put in a desiccator to completely evaporate HFIP, and thereafter stored at −80 °C. Oligomer solutions were prepared freshly for each experiment. The stock was dissolved in 10 μL of DMSO (to 105 μM) and incubated for 3 h at room temperature. Oligomers of Aβ(1-42) were added to the day-30 ChP organoid cultures derived from human iPSK3 cells at 1 μM for three days. The cells were harvested for mRNA isolation and RT-qPCR.

### 4.9. Extracellular Vesicle Isolation

For EV isolation experiments, culture media were replaced by EV-depleted media (using FBS depleted of EVs by ultracentrifugation) during days 30–38. Conditioned media were sequentially spun (500× *g* for 5 min, 2000× *g* for 10 min, 10,000× *g* for 30 min) to remove cell debris, apoptotic body, large vesicles, etc. Polyethylene glycol (PEG)-6000 was added to the supernatant to a final end-concentration of 8% (*w*/*v*) PEG in 0.5M NaCl and stored for 24 h at 4 °C as previously described [20,58,59]. The suspension was spun at 3000× *g* for one hour and the supernatant was discarded. The remaining pellet was suspended in 1 mL PBS and ultracentrifuged at 120,000 × *g* for 70 min at 4 °C. The EV pellet was then re-suspended in 200 µL PBS using a benchtop shaker at 1500 rpm for 5 min. EVs were diluted to 10^8^–10^9^ particles per mL in PBS for further characterizations [59,60].

### 4.10. Nanoparticle Tracking Analysis (NTA)

The particle size distribution and zeta potential of isolated EVs were assessed using a nanoparticle tracking analysis (NTA) and a Zeta View instrument (ZetaView^®^ TWIN PMX-220, Particle Metrix, Ammersee, Germany), respectively, which utilizes the dynamic light scattering (DLS) technique at 25 °C with a 90° scattering angle. Zeta-View Analysis software was used for data processing as previously described (all the EV samples were prepared by diluting 1:1000 with PBS (particle-free)) [60,61].

NTA was also preformed using a Nanosight LM10-HS instrument (Malvern Instruments, Malvern, UK). It is configured with a blue laser (488 nm) and sCMOS camera. The samples were diluted as 1:1000 in filtered PBS. Three videos of 60 s were captured with a camera shutter speed fixed at 30.00 ms. The camera level was set to 13, and the detection threshold was set to 5. Between each sample reading, the laser chamber was cleaned thoroughly with particle-free milliQ. The collected videos were analyzed using NTA3.4 software to obtain the mode and mean size distributions, as well as the concentration of particles.

### 4.11. Transmission Electron Microscopy (TEM)

TEM was performed to confirm the morphology of EVs according to Lasser et al. [62] and our previous publication [20]. Briefly, EV isolates were resuspended in 50–100 μL of sterile filtered PBS. For each sample preparation, intact EVs (5 µL) were dropped onto Parafilm. A carbon-coated 400 hex mesh copper grid (Electron Microscopy Sciences, EMS) was positioned using forceps with the coating side down on top of each drop for one hour. Grids were washed with sterile filtered PBS three times and then the EV samples were fixed for 10 min in 2% PFA (EMS, EM Grade). After washing, the grids were transferred on top of a 20 µL drop of 2.5% glutaraldehyde (EMS, EM Grade) and incubated for 10 min at room temperature. Grid samples were stained for 10 min with 2% uranyl acetate (EMS grade). Then, the samples were embedded for 10 min with 0.13% methyl cellulose and 0.4% uranyl acetate. The coated side of the grids were left to dry before imaging on the CM120 Biotwin electron microscope [62].

### 4.12. Statistical Analysis

Representative experiments are presented, and the results are expressed as mean ± standard deviation. To assess the statistical significance, a Student’s *t*-test and a one-way ANOVA followed by Fisher’s LSD post hoc tests were performed. A *p*-value < 0.05 was considered statistically significant.

## 5. Conclusions

This study derived ChP organoids from hiPSCs in the presence and absence of Wnt activation. The Wnt activation upregulated most of the ChP markers, and the derived ChP organoids were more responsive to the Aβ42 oligomer stimulation with the increased *TNFα*, *IL-6*, and *MMP2*/*9* expression. The EV biogenesis was more affected by the ChP organoid development time than the extent of the Wnt activation. The released EVs at day 30 had a comparable size distribution, zeta potential, and a higher particle concentration for the Wnt-activated condition. This study provides new knowledge related to the inflammatory response and the EV biogenesis of hiPSC-derived ChP organoids. In addition, this study provides a physiologically relevant model for disease modeling and drug screening investigations to assess treatments for neurological disorders.

## Data Availability

The data presented in this study are available on request from the corresponding author.

## Supplementary Materials

The following supporting information can be downloaded at: https://www.mdpi.com/article/10.3390/ijms24087660/s1.
