# Supplementary material for: Inflammatory Response and Exosome Biogenesis of Choroid Plexus Organoids Derived from Human Pluripotent Stem Cells
Source: Int J Mol Sci. 2023 Apr 21;24(8):7660. doi: 10.3390/ijms24087660 (PMC10146825; doi:10.3390/ijms24087660)
Supplement: Supplementary file 1 [file ijms-24-07660-s001.zip › ijms-2336520-supplementary.pdf]

Supplemental Materials

Inflammatory Response and Exosome Biogenesis of Choroid Plexus Organoids Derived from Human Pluripotent Stem Cells

Laureana Muok[1], Chang Liu[1], Xingchi Chen[1], Colin Esmonde[1], Peggy Arthur[2], Xueju Wang[3], Mandip Singh[2], Tristan Driscoll[1], Yan Li[1,*]

1. Department of Chemical and Biomedical Engineering, FAMU-FSU College of Engineering, Florida State University, Tallahassee, FL 32310, USA
2. College of Pharmacy and Pharmaceutical Sciences, Florida A&M University, Tallahassee, FL 32307, USA
3. Department of Materials Science and Engineering, University of Connecticut, Storrs, CT 06268, USA

* Correspondence: yli4@fsu.edu

Supplemental Figure S1. The aggregate size image analysis (day 3, 7, 11, 16, 21, 28). The growth conditions were the same for CHIR+ and CHIR- conditions during day 0-11 before CHIR was added to the culture media.

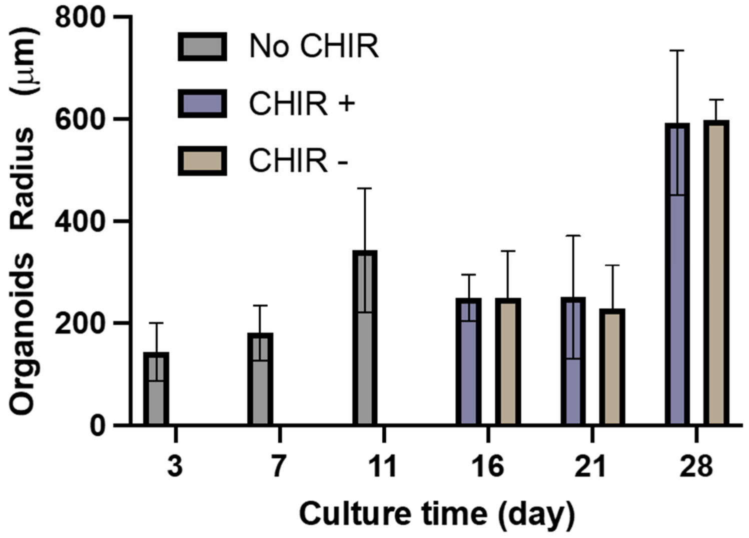

Supplemental Figure S2. The amount of mRNA isolated for CHIR- and CHIR+ conditions. The differentiation was initiated with the same number of the cells for the two conditions. The difference in spheroid number was evident after CHIR was added to the culture compared to no CHIR was used. The organoids from the same number of wells were used for mRNA isolation, which should come from the same number of cells at day 0. The results from three experiments were shown (Exp 1, 2, and 3).

Exp 1

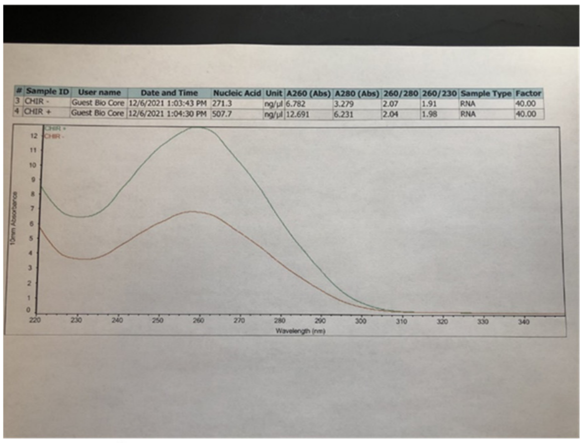

#	Sample ID	User name	Date and Time	Nucleic Acid	Unit	A260 (Abs)	A280 (Abs)	260/280	260/230	Sample Type	Factor
3	CHIR -	Guest Bio Core	12/6/2021 1:03:43 PM	271.3	ng/µl	6.782	3.279	2.07	1.91	RNA	40.00
4	CHIR +	Guest Bio Core	12/6/2021 1:04:30 PM	507.7	ng/µl	12.691	6.231	2.04	1.98	RNA	40.00

Exp 2

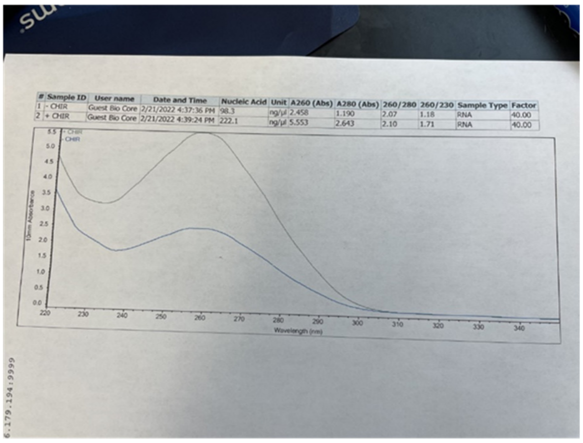

#	Sample ID	User name	Date and Time	Nucleic Acid	Unit	A260 (Abs)	A280 (Abs)	260/280	260/230	Sample Type	Factor
1	- CHIR	Guest Bio Core	2/21/2022 4:37:36 PM	98.3	ng/µl	2.458	1.190	2.07	1.18	RNA	40.00
2	+ CHIR	Guest Bio Core	2/21/2022 4:39:24 PM	222.1	ng/µl	5.553	2.643	2.10	1.71	RNA	40.00

Exp 3

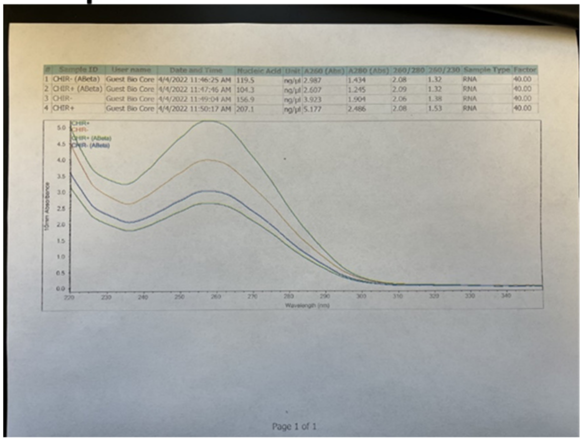

#	Sample ID	User name	Date and Time	Nucleic Acid	Unit	A260 (Abs)	A280 (Abs)	260/280	260/230	Sample Type	Factor
1	CHIR- (ABeta)	Guest Bio Core	4/4/2022 11:46:25 AM	119.5	ng/µl	2.987	1.434	2.08	1.32	RNA	40.00
2	CHIR+ (ABeta)	Guest Bio Core	4/4/2022 11:47:46 AM	104.3	ng/µl	2.607	1.245	2.09	1.32	RNA	40.00
3	CHIR-	Guest Bio Core	4/4/2022 11:49:04 AM	156.9	ng/µl	3.923	1.904	2.06	1.38	RNA	40.00
4	CHIR+	Guest Bio Core	4/4/2022 11:50:17 AM	207.1	ng/µl	5.177	2.486	2.08	1.53	RNA	40.00

Isolated mRNA	CHIP+/CHIR- ratio
Exp 1	1.87
Exp 2	2.26
Exp 3	1.32
Mean±SD	1.82±0.47
Exp 3-Abeta	0.87

Supplemental Figure S3. The Live/Dead assay images for the replated organoids to support Figure 2A. The scale bar: 50 µm.

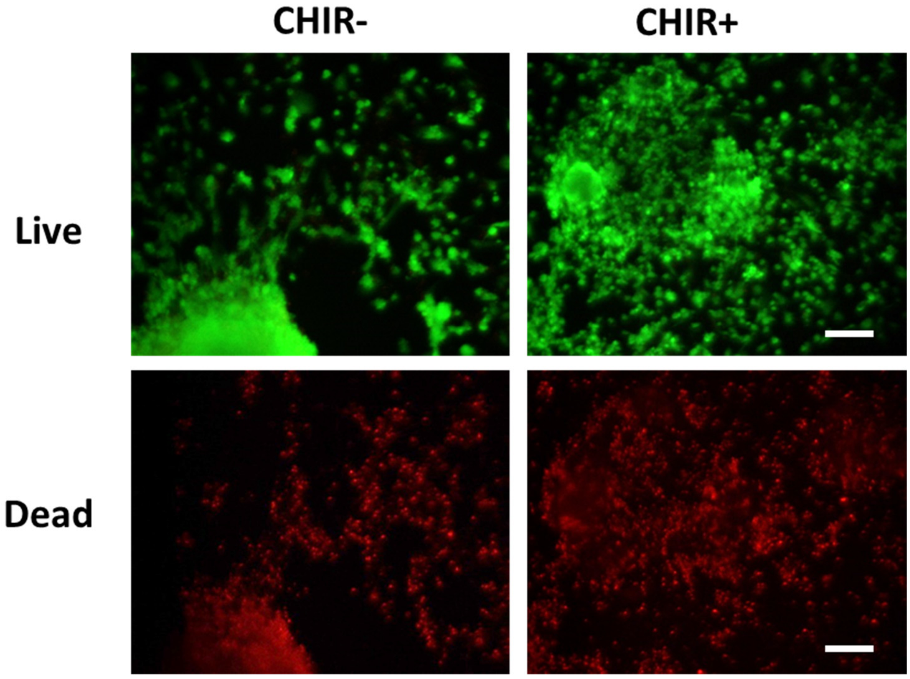

Supplemental Figure S4. Example of SSC vs. FSC plot to show flow cytometry gating strategy for Figure 2.

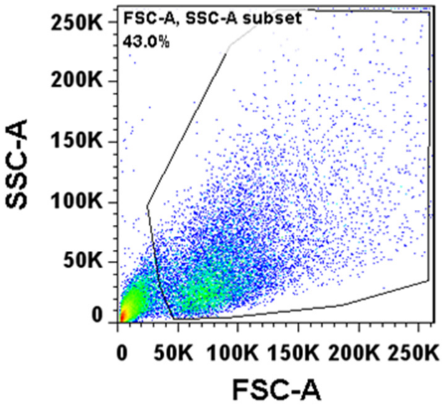

Supplemental Figure S5. The schematic illustration of extracellular vesicle isolation procedure. Christopher Jean-Baptiste prepared the schematic illustration of exosome isolation using BioRender.

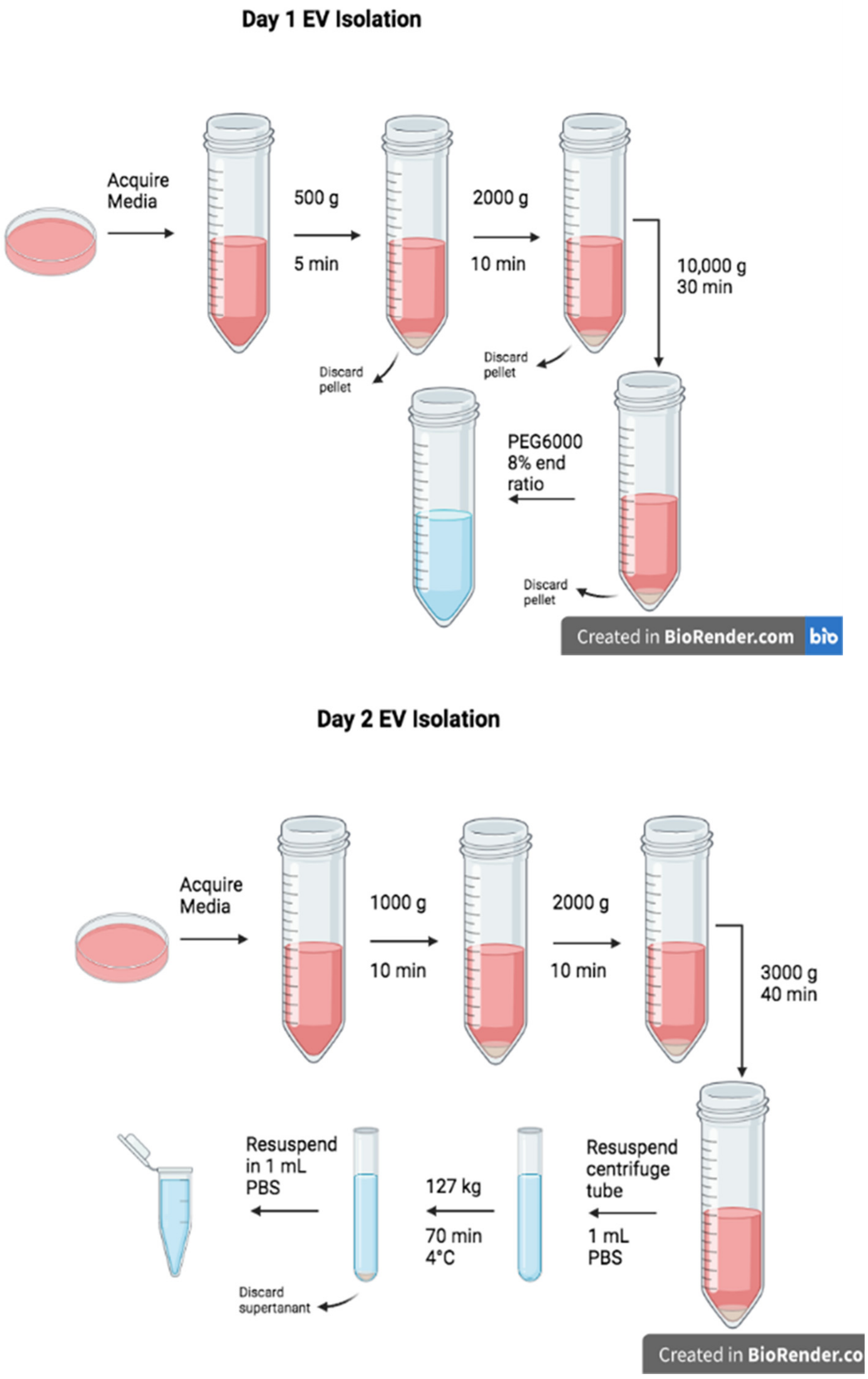

Supplemental Figure S6. Zeta potential of ChP organoid-secreted extracellular vesicles determined by ZetaView.

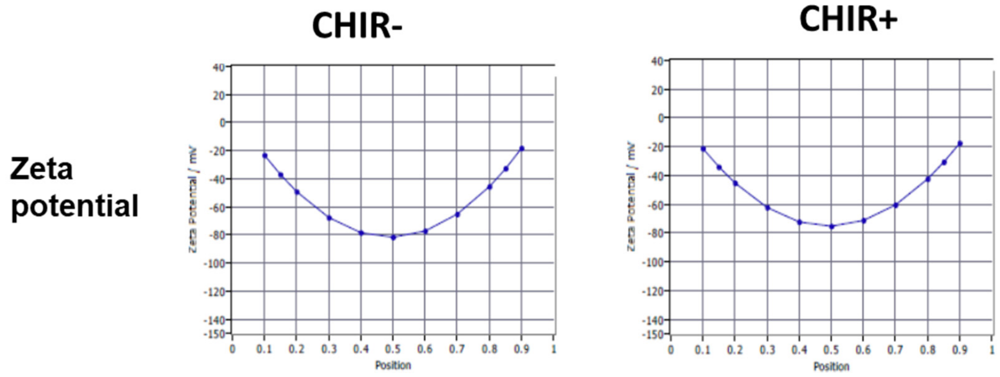

Supplemental Table S1. Normalization of EV number to the isolated mRNA amount.

Parameters	CHIR-	CHIR+	CHIR+/CHIR- ratio
**EV number**	7.8±2.6×10^7 per mL	12.6±2.7×10^7 per mL	1.62
**mRNA amount**	271 ng/µL (mg/mL)	508 ng/µL (mg/mL)	1.87
**EV/mRNA**	2.88±0.96×10^5 /mg	2.48±0.53×10^5 /mg	**0.86±0.15**

Supplemental Table S2. ChP marker analysis for mRNA-Seq in Song et al (2019) study [39].

Transcriptome Analysis of ChP Markers in cortical organoids. 3D forebrain organoids containing isogenic microglia-like cells (DMG) vs. microglia-like cells only (MG). the changes are expressed as Log2(DMG/MG). A positive value means upregulation in DMG group.

The meaning of these markers are explained in the main text.

GeneName	baseMean	log2FoldChange
**DCN**	32827.22	**3.1730**
**LUM**	35876.97	**2.7775**
**IGFBP7**	232.5938	**2.2776**
**TTR**	2570.966	**1.7943**
**CLIC6**	23.89272	**1.6367**
**MSX1**	1182.936	**1.4409**
**PLTP**	7230.866	**1.4052**
**AQP1**	30424.8	**1.1713**
**DLK1**	5533.424	**0.8398**
**PLEC**	7382.984	**0.4296**

The expression of tight junction markers: TJP1/2, CLDNs, OCLN, MPDZ; specific transporters involved in nutrient trafficking: SLC46A1, SLC23A2. Clinically relevant markers: SERPINF1-serpin family F member; PARK7-Parkinsonism associated deglycase. OTX2- early transcription factor involved in ChP development.

GeneName	baseMean	log2FoldChange
**SERPINF1**	3889.831	**2.3839**
**TJP2**	726.8553	**1.6434**
**SLC46A1**	170.2765	**1.5218**
**TJP1**	1701.744	**0.7481**
**CLDN5**	23.0293	**0.5514**
**OCLN**	161.7074	**0.1442**
**PARK7**	2734.893	**0.0742**
**RSPO3**	755.9327	**0.0302**
**INADL**	505.462	**0.0231**
**MPDZ**	1256.236	**-0.1027**
**SLC23A2**	1138.679	**-0.1837**
**CLDN3**	91.25794	**-0.5217**
**CLDN1**	120.5591	**-1.4768**
**CA2**	343.5995	**-2.1864**
**HTR2C**	27.34546	**-6.2390**
**OTX2**	689.1214	**-9.1203**

Ciliary markers-CCDC67, ARL13B; Ciliary transcription factor: FOXJ1

Brain-derived factor-secretogranin-1 (CHGB), a neuro-endocrine secretory protein; NDRG2, an astrocyte-expressed protein that plays a role in neurite growth. C1R-complement protein. KRT17-keratin 17; TAGLIN-transgelin, expressed in a subset of cells in epithelium.

RBPs: retinol binding proteins, the more mature markers expressed in dark cells of ChP epithelium. IGF2: restricted in dark cells of ChP epithelium.

LGALS3BP: human-specific developmental secreted protein.

GeneName	baseMean	log2FoldChange
**CHGB**	1920.537	**2.4160**
**C1R**	737.2601	**2.3750**
**NDRG2**	1456.452	**1.5622**
**CCDC67**	9.219291	**0.9642**
TAGLN	568.3037	0.9241
**ARL13B**	222.5634	**0.1401**
KRT17	5.476122	-0.4263
**FOXJ1**	138.8025	**-4.1214**
**GeneName**	**baseMean**	**log2FoldChange**
**RBP4**	661.1704	**2.5971**
**MDK**	11336.73	**2.3416**
**RBP3**	29.90876	**2.2582**
**LGALS3BP**	6305.362	**2.2219**
**IGF2**	37271.79	**2.1672**
**RBP2**	68.89518	**1.2927**
**RBP1**	557.7267	**0.9952**
**PRAP1**	10.72332	**0.0833**

Supplemental Table S3. A list of antibodies.

Cells	Primary Antibody	Origin/ Isotype	Supplier/ Cat#	Dilution
ChP markers	TTR	Mouse IgG1	Abcam, ab204997	1:200
	CLIC6	Rabbit IgG	Abcam, ab204566	1:500
Neuronal cells	Beta-tubulin III	Mouse IgG1	Millipore, MAB1637	1:100
Secondary	Alexa Fluor 488, goat anti-mouse IgG1	-	ThermoFisher, A-21121	1:200
	Alexa Fluor 488 Goat Anti-rabbit IgG		ThermoFisher, A-11008	1:200

Supplemental Table S4. Primer sequence for the target genes determined by RT-PCR.

Primer number	Gene name	Primer name in database	Primer sequence (5'-3')
1	ACTB	Bactin F	GTACTCCGTGTGGATCGGCG
		Bactin R	AAGCATTTGCGGTGGACGATGG
2	GAPDH	GAPDH-F	TCACTGCCACCCAGAAGACTG
		GAPDH-R	GGATGACCTTGCCCACAGC
3	CLIC6	CLIC6-F1	GCAGTTTTACCCCAGCAGTCA
		CLIC6-R1	AATCTGCCCCCTAACCAGATG
4	PLEC	PLEC-F1	CTGTAGAGGGCCCTGGTGTTT
		PLEC-R1	GGGGCTTCTCTGGGTAGACTG
5	PLTP	PLTP-F1	CCCTGACTGAGAGGAACTGGA
		PLTP-R1	TCCATGGCAGAGTCGAAGAAG
6	TTR	TTR-F1	TGGGAAAACCAGTGAGTCTGG
		TTR-R1	GATGCCAAGTGCCTTCCAGTA
7	IGFBP7	IGFBP7-F1	GGAACAAGGTAAAAAGGGGTCA
		IGFBP7-R1	AGCACCCAGCCAGTTACTTCA
8	MSX1	MSX1-F1	GCCTTCCCTTTAACCCTCACA
		MSX1-R1	GGGACTCTTCCAGCCACTTTT
9	DCN	DCN-F1	GATTTGGTGGGGAGGCACTAT
		DCN-R1	GCCTCCTTTATGCCAACCTGT
10	LUM	LUM-F1	GGCAGGCCTATTTCATCACAA
		LUM-R1	AAGGTTTTGCACATCATTTGACAG
11	DLK	DLK1-F1	CTGAGAGTGTCGGGATCCTTG
		DLK1-R1	TGGATCGCTGTCTTTGAGCTT
12	AQP1	AQP1-F1	CAGCCCAAGGACAGTTCAGAG
		AQP1-R1	TCATGGCTAAGTGCACAGTGG
13	TNFa	Forward	TGGCCAATGGCGTGGAGCTG
		Reverse	GTAGGAGACGGCGATGCGGC
14	IL-6	Forward	GAACTCCTTCTCCACAAGCG
		Reverse	TTTTCTGCCAGTGCCTCTTT
15	IL-12β	Forward	CCAAGGGGTGACGTGCGGAG
		Reverse	GGTGGGTCAGGTTTGATGATGTCCC
16	CD163	Forward	CCAGTCCCAAACACTGTCCT
		Reverse	ATGCCAGTGAGCTTCCCGTTCAGC
17	TGFβ	Forward	CCTACATTTGGAGCCTGGAC
		Reverse	TGTCCTTAAATACAGCCCCC
18	IL-10	Forward	AAGCCTGACCACGCTTTCTA
		Reverse	ATGAAGTGGTTGGGGAATGA
19	SMPD2-1	SMPD2-F1	GCCTGGGAGACTTTCTGAACC
		SMPD2-R1	AAGTGGTGTGCAGCTGGGTAG
20	SRSF5(hrs)-1	SRSF5-F1	CTTCTCGGATCGAGGCTTCTT
		SRSF5-R1	TCGAATCAACTGCGCTCATTA

21	TSG101	TSG101 F	CACCTGGTGGTCCATATCCTG
		TSG101 R	GATGGTGTCCTCGCTGATTGT
22	STAM1	STAM1-F1	CACTGGATTTTTGGGTTGCTC
		STAM1-R1	GTGGAAAACATTTTTCGCATGA
23	PDCD61P (ALIX)	PDCD61P F	TAAGTGCATCTGAGGGCCAAA
		PDCD61P R	GGGGCCTCCTTTCCTAGTTTC
24	PDCD61PI4 (ALIXI4)	PDCD61Pi4 F	TTGGCTAATCAGGCTGCAGAT
		PDCD61Pi4 R	TCACATGCAAAGTAAGCAAGTGTT
25	MITF-1	MITF-F1	GAATTGGTGATGGGTGATGGA
		MITF-R1	TGCATGGGAACTATGCAGTTG
26	RAB27B	RAB27B F	TCCATGAAGCTGCTTGTCTCA
		RAB27B R	GTTGGGTCTCCACCCAGAAAT
27	MMP2	MMP2 F1	CATCGCTCAGATCCGTGGTG
		MMP2 R1	GCATCAATCTTTTCCGGGAGC
28	MMP3	MMP3 F1	CCATCTCTTCCTTCAGGCGT
		MMP3 R1	ATGCCTCTTGGGTATCCAGC
29	MMP9	MMP9-F	GGCCACTACTGTGCCTTTGAG
		MMP9-R	AATCGCCAGTACTTCCCATCC
